# Exploring the care needs of Iranian patients with cancer: a qualitative content analysis

**DOI:** 10.1186/s12912-021-00659-3

**Published:** 2021-08-09

**Authors:** Fariba Mazhari, Zohreh Khoshnood

**Affiliations:** 1 Department of Fundamental Nursing, Geriatric Care Research Center, Faculty of Nursing and Midwifery, Rafsanjan University of Medical Sciences, Rafsanjan, Iran; 2 Nursing Research Center, Razi School Faculty of Nursing and Midwifery, Kerman University of Medical Sciences, Kerman, Iran

**Keywords:** Cancer, Iran, Care needs, Qualitative study

## Abstract

**Background‏:**

Cancer is currently one of the most critical health problems worldwide. Different studies have shown that disease can reduce people’s ability to take care of themselves and this makes them face many needs. Therefore, this study aimed to investigate unmet needs faced by patients with cancer‏.

**Methods:**

This study employed a conventional, qualitative content analysis method. Data saturation was achieved after interviewing 13 participants in 15 interview sessions. With the in-depth semi-structured approach, the participants were asked to narrate their experiences of self-care needs in the process of disease. The collected data were analyzed using Graneheim and Lundman’s method through the following steps: Construction of the units of analysis, construction of codes of meaning, condensation, extracting codes, and categorization (subcategories, categories, and the core category) ‏.

**Results‏:**

Data analysis revealed two main categories. The first category was “Deficiency in disease management” with three subcategories: “the need to get rid of annoying physical symptoms”,“ the need for a caregiver to help with the daily routine”, and “feeling frustrated and giving up treatment”. The second main category covered “the need for sympathetic and knowledgeable caregivers” with the following five subcategories:” the need for sympathy and interaction with the nurse”, “the need to hear the truth about the illness “, “the need for appropriate therapeutic interaction from physicians”, “Inadequate qualification of medical secretaries about the patient”, and” need for advice from psychologists for psychological adaptation‏”.

**Conclusion‏:**

The most critical need reported by the patients was the need for compassionate and informed caregivers. So nursing management and care with kindness, competence, and conscience is the fundamental right of patients with cancer. Identifying patients’ needs and problems can be used as a background for increasing the knowledge and experience of nurses and providing care for patients with cancer‏.

## Background

Cancer is a major health problem worldwide, especially in developing countries. It is also the leading cause of death in the world and the third leading cause of death in Iran [1]. According to the Globocan 2018 estimates, the incidence and deaths of cancer in Asia are 48.4 and 57.3 %, respectively [2, 3]. Iran, as a developing country, has been faced with a growing trend of cancer deaths especially due to the population aging and an increase in cancer risk factors during the recent decades [4] After traffic accident and cardiovascular mortality, cancer was suggested as the third leading cause of death in Iran [4, 5]. Overall, 112,060 new cancer cases (including skin cancers) were registered in INPCR in 2014 [6].

Cancer can reduce people’s ability to do routine work followed by feelings of depression, frustration, and other psychological problems [7–9]. Previous studies have shown that the highest level of support needs is self-care for meeting cancer patients’ physical needs and helping them to adapt to illness [10]. Research has also suggested that the most critical primary needs of patients with cancer were a deficiency in daily care, fatigue, pain, and the lack of advice from the treatment staff [11]. Following this, self-care is defined as one of the most effective methods for controlling the physical and psychological effects of the disease [9]. According to Orem, self-care can cover all activities for the protection of life, health, and well-being [12, 13]. Therefore, it can be stated that self-care includes all activities that are undertaken for prevention, treatment, and increasing the quality of life, and satisfaction of the person with experience and rehabilitation [14].

The analysis of patients’ experiences shows that nursing care is one of the critical indicators in treatment and improving the quality of patient care [15], which largely depends on the quality of the communication with the patient [16]. Lack of proper control of cancer complications exacerbates the negative effects of the disease on patients’ quality of life. This highlights cancer patients’ need for comprehensive physical, psychological and social care so that they can have a quality life [17]. Therefore, from the perspective of patients with cancer, nurses have an essential role in recognizing self-care needs and providing emotional and psychological care. Accordingly, counseling and support must be provided for people with cancer and their families at all stages of the disease. Furthermore, patients should be assured that they will receive the best care, and they will be assisted to take care of themselves [18].

Cancer patients’ ability of self-care is important as shown by other studies that have addressed patients’ care needs. Besides, rehabilitating such patients and helping them with their daily activities can make a significant contribution to reducing the psychological complications of the disease. One of the developmental tasks of patients with cancer is to take care of themselves. However, cancer survivors have special self-care needs [9]. Given the complexity, ambiguity, and multidimensionality of self-care needs, this study aimed to clarify the dimensions and characteristics of care needs of in a group of patients with cancer through a qualitative approach.

## Methods

### Ethics approval statement

The proposal of the present study was approved under the code of ethics IR.KMU.REC.1398.464 by the Ethics Committee of Kerman University of Medical Sciences. A written consent form was obtained from all participants. The researcher explained the research procedure to the participants and informed them that they could leave the study at any time. They were also ensured that no names or facts were to be stated in the data. Speaking about their needs and the experiences related to this subject provokes emotions and may be a painful reminder of various situations. The researchers handled this risk by being attentive and sensitive to the interviewees’ emotional reactions.

### Data collection

This study was conducted as a conventional qualitative content analysis with a descriptive explorative approach. The data were collected from 13 participants including 10 patients with cancer, two nurses, and a family member. The data were saturated after interviewing 13 participants in 15 sessions. Two participants were interviewed two times. To increase the theoretical sensitivity, theoretical sampling was performed. Thus, patients, family members, and nurses with different and rich experiences were invited to the interviews. Data analysis was performed separately in each group of samples and finally, they were classified based on similarities and differences. Besides, individuals with different characteristics in terms of age, role, and work experience were chosen by the second researcher to provide a wide range of information. To achieve maximum data saturation and to ensure patients’ experiences, their family members and nurses were interviewed to collect data. On average, the patients were suffering from cancer for nine months to four years. The participants’ mean age was 45.7 years (Table 1). The patients were interviewed‏‏ from autumn 2016 to spring 2017‏‏. They referred to three hospitals affiliated with Kerman University of Medical Sciences to use medical services. A purposive sample of patients who had an experience of cancer participated in the research.

**Table 1:** characteristic of participants

Participant‘s code	Age	Gender	Cancer type	Marital status
1	34	Male	Leukemia	Single
2	47	Female	Breast	Married
3	29	Male	Leukemia	Single
4	55	Female	Breast	Married
5	44	Female	Breast	Married
6	40	Female	Breast	Divorced
7	70	Male	Colon	Married
8	56	Female	Breast	Married
9	37	Female	Pancreatic	Married
10	45	female	Non-Hodgkin’s Lymphoma	Married
11	38	female	Nurse with 9 years’ work experience in the oncology ward	Married
12	34	female	Nurse with 10 years’ work experience in the oncology ward	Married
13	37	female	Mother of a patient with Leukemia	Married

In-depth individual semi-structured interviews were conducted with the participants at their preferred time and places. The participants were asked to narrate their experiences of their care needs. Probing questions were also asked for clarification: ‘Please, explain more about your care needs when your disease started?‘, ‘What do you do when you feel the need for a care?‘; ‘Can you provide an example’? The interviews were tape-recorded, transcribed verbatim, and analyzed by the first author. Each interview lasted between 40 and 110 min.

### Data analysis and interpretation

The collected data were analyzed using Graneheim and Lundman’s method through the following steps: Construction of the units of analysis, construction of codes of meaning, condensation, extracting codes, and categorization (subcategories, categories, and the core category)‏.In this study, each interview was considered a unit of analysis. Afterward, the text was divided into meaning units. Each meaning unit consists of words, sentences, or paragraphs containing aspects related to each other through their content and context. In the next step, the meaning units were condensed, while still preserving the core. The condensed meaning units were then labeled with a code and subcategories were created. The next step was to create categories that represent the core feature of qualitative content analysis. A category is a group of codes that are similar at a manifest level. The main category is a recurrent thread of underlying meaning running through codes and categories; it can be seen as an expression of the latent meaning of a text [19]. Although the analysis process was systematic, it was a back-and-forth movement between the whole and parts of the text. Sampling continued until the data were saturated and no new information was extracted.

Several techniques were used to enhance the credibility of the current study. The second researcher’s supervisors have done peer checking. Through frequent sessions between the second researcher and the supervisors, the study’s progress and process were reported and discussed. Member checking was completed with some of the participants for validation of interpreted findings. Some of the faculty members checked the encoding process and access to categories. Also, a clear and detailed description of the culture, context, selection, and characteristics of participants, data collection, and process of analysis was provided.

## Results

Data analysis leads to the extraction of the two main categories including the “deficiency in disease management with three subcategories and “the need for sympathetic and knowledgeable caregivers“with the five subcategories.

### Deficiency in disease management

At the onset of cancer and the progression of its symptoms, patients are faced with multiple physical difficulties and complications that are considered as basic needs of these patients. In this study, the patients stated they felt unable to manage their symptoms and complications and they needed help.

### Need to get rid of annoying physical symptoms

Among the most critical problems reported by the patients were complications from chemotherapy. The patients reported that they faced many challenges to overcome these complications and sometimes found themselves close to death. During the chemotherapy process, the patients had several experiences:

*“Chemotherapy is like putting a bomb inside my body and then pressing its button. I have high pain tolerance, but when my white blood cells go down, I feel terrible. It’s so painful. (P10) »*.

### Need for a caregiver in daily routine

The majority of patients, after the onset of the disease and particularly during treatment, stated that they were concerned about their daily routine, the fear of losing independence, the need for a caretaker to do their daily activities, and the failure to do things that they used to do before.

*“When I was hospitalized and had chemotherapy, it was tough for me to pick up or move something and I needed someone to do it for me (p6)”*.

*“The side-effects after chemotherapy are considerably tough. The patient suffers from these complications, and because of that, he/she becomes overwhelmed and disabled. (P11 Oncology Nurse)”*.

### Feeling frustrated and giving up treatment

When the patient is suffering from frequent hospitalizations, he/she becomes disconcerted and, when his/her treatment fails, he/she feels frustrated, and he/she is more likely to give up the treatment.

*“It’s an unfortunate thing when his mood breaks down, and he says I’m not going to get well. I’ll die. Who has recovered well from this illness? (P13.The mother of a patient)*

### Need for sympathetic and knowledgeable caregivers

According to the experiences of participants, this category included four subcategories:

- Need for sympathy and interaction with a nurse

The participants in this study stated that empathy and the nurse-patient relationship as one of the main criteria for care. Some participants were satisfied with this relationship. They considered the treatment team as an essential factor in their treatment and recovery. However, some participants were dissatisfied with this relationship and stated that the nurses lacked knowledge and competence in oncology weak and needed further nursing education.

*“Those times I was eating my soup like a cat because I could not move my hands. The nurse asked if she could help me to put it in my mouth. I said no. She sat on the chair next to me. Suddenly I saw her crying. It was a good sense of empathy for me” (P3).*

*“New nurses should receive training, but that’s not enough. Nurses need more hands-on expertise and skills” (P11 oncology nurse).*

- Need for hearing the truth about the disease

The cancer patients in this study reported that from the moment they were diagnosed with the disease, thousands of ambiguous questions about the nature of the disorder come to their mind, so they needed reliable information for an informed person to clarify the issues in their minds. Some of the patients said that hearing the truth would help them to enter the stage of “disease acceptance” and fight their disease.

*“I needed a person to explain to me what this disease was overall. How does it happen? Because this disease is like a giant that creates fear and horror (P1).“*

*“The doctor told me, 0.7 mm of the tumor remained after the operation, and there was a significant danger. I liked her honesty because she did not hide my illness (p8). “*

- Need for appropriate therapeutic interaction from physicians

The participants highlighted the need for establishing a productive therapeutic relationship between the doctor and the patient in the process of treatment. Many patients stated that if a person is diagnosed with cancer, he or she should first be mentally prepared.

*“When I received my test results, the nurse said, “Madam, you have cancer, you need chemotherapy.“ As soon as she said this, I fainted and fell to the ground. She didn’t try to prepare me mentally and then tell me (P4). “*

### Inadequate qualification of medical secretaries about the patient

One of the problems, as the participants stated, was medical secretaries’ mistreatment of the patients. Therefore, the participants believed that the secretaries needed to be trained and informed about cancer patients’ psychological and physical conditions.

*“The secretaries should also be trained. They are so bad-tempered, and they want to fight. I even saw several times that they started fighting with the patients’ companion (P10).”*

- Need for advice from a psychologist for psychological adaptation

Since the onset of cancer and the psychological crisis, patients may not have the ability to communicate appropriately and accept the condition. Therefore, they need counseling and advice from an informed person. Unfortunately, there is currently no provision of psychological services in oncology departments, and this burden has been imposed on nurses.

*“We don’t have psychoanalysts in our wards. There are some cases in which the patient has a terrible psychological state and needs advice from a psychologist (P12; an oncology nurse)”.*

## Discussion

The present study showed that cancer patients faced many problems in the management of the disease and its complications. Studies have shown that cancer can reduce people’s ability to do routine work, followed by feelings of depression, frustration, and other psychological problems [17, 20]. For instance, Jansen (2015) reported that the overall patients’ understanding of self-care is mostly related to physical self-care problems, the need for counseling, the need for social care, the need for psychological care, and finding a solution to their life problems [21]. Furthermore, Moghaddam et al. (2016), O’Brien et al. (2017), and Boyes et al. (2012) reported that the most critical primary needs of patients with cancer were their inability to do daily care, fatigue, pain, and the need for counseling with the staff treatment [10, 11, 22].

The present study showed that the feeling of frustration and the need to get rid of it in patients led to the deficiency in disease management. Davis (2017), and Chu-Hui (2007) found that hope is one of the essential elements in the life of patients with cancer [23, 24]. Several factors can help patients feel hopeful. People’s demographic characteristics, especially age and sex, mental status, social environment, spiritual or religious beliefs, symptoms and complications of the disease, and previous experiences may affect their hopefulness and ability to take care of themselves [21, 23, 25, 26]. Previous studies (Davis, 2017; Schjolberg, 2011; Jakobsson, 2015) reported that there is a direct relationship between mental and physical problems, weakness, pain, and a sense of hope and well-being in patients with cancer [23, 26, 27]. According to various studies, this sense of confidence can increase by focusing on self-control, self-awareness, self-esteem, and a greater understanding of satisfaction with life [26, 28]. According to the participant’s statements, they were able to more easily deal with the physical and emotional problems caused by the disease by focusing more on self-knowledge.

One of the patients’ needs was to have access to a source for getting information on their illness. Stahl (2017) stated that one of the least rights of the patients is the right of knowing and awareness. One of the essential duties of physicians is to promote patients’ independence. Thus, respect for the right to “know” is respect for patients’ autonomy and authority. When patients receive accurate information about their illness and its prognosis, they can take realistic actions about self-care and disease management [29]. Papadakos (2017), Giuliani et al. (2016), and Manne (2016) highlighted the need for information in cancer patients about physical care, medical information, and information to meet their emotional, social, and spiritual needs [30–32]. A systematic review by Moghaddam et al. (2016) suggested that four primary unmet needs in patients with cancer include a lack of awareness, concern, and uncertainty about the future of the illness, fatigue, and deficiency in the disease management [11].

One of the essential caring needs in this study was a deficiency in communication and the training of the patient by physicians, nurses, other members of the treatment team. Nursing care is defined in terms of four physical, psychological, social, and spiritual areas. Therefore, nursing care is one of the critical factors in the treatment and improvement of care quality [15]. This care depends on the quality of communication with the patient [16]. For improving the quality of care psychological, functional, self-care, and financial needs must be considered. Cooperation among the treatment team members, including the nurse and the patient, as well as home follow-up care, should also be taken into account [15].

Nurses must have the expertise, competence, knowledge, and skills in providing care and support services to patients [14]. Makarem et al. (2016) stated that nursing care in Iran is influenced by economic, social, political, and cultural issues [33]. Rafii et al. (2008) reported that heavy workload, shortage of workforce, and nurses’ limited authority reduced patient satisfaction [34].

In the current study, one of the patients’ needs was a deficiency in communication with physicians. Herd (2014) and Stefan (2010) reported that communicating with physicians and receiving clear answers from physicians as well as spiritual support by them plays a vital role in reducing anxiety and increasing patient satisfaction with care provision [35, 36]. Contrary to the current study, Makarem et al. (2016) found the highest level of patient satisfaction with physicians’ actions (77.8 %) was due to an observance of ethical standards (76 %) [33]. The difference between the results of the two studies can be attributed to the methodological differences in these studies and also the differences in the provision of care and treatment and physicians’ therapeutic culture.

An exploration of the participants’ experiences in the current study showed that the unavailability of psychologists is one of the primary defects in the oncology wards. Unfortunately, in … province, patients’ need for counseling has not been met, and patients are always struggling with their psychological problems. Likewise, Jansen et al. (2015) and Moghaddam et al. (2016) stated that cancer patients’ primary need was the need for counseling with the treatment team [11, 21].

## Strengths and limitations

The strengths of this study include an obvious and detailed description of the culture, context, selection, and characteristics of participants, data collection, and technique of analysis. Subjectivity could likely have been of influence in identifying the themes. However, we tried to minimize the possibility because the data were transcribed verbatim, and checked by some of the faculty members the encoding process and access to categories. We also attempted to minimize the influence of subjectivity by seeking consensus. By using the purposive sampling method, we planned to include all eligible patients during the period of data collection. Still, a small number of eligible patients might not have been identified quickly enough for researchers to be on time for the admission interview. Therefore, some relevant data may have been missed.

## Conclusions

The present study identified care needs in patients with cancer. The essential demands from the patients were the requirements for the management of the disease and the need to have caregivers. Accordingly, patients need care with kindness, competence, and conscience, which is the fundamental right of patients with cancer and their families. One of the tasks of the treatment team is to empower cancer patients to engage in self-care behaviors. This can reduce the complications of the disease. Besides, given the increase in the number of cancer patients and patients’ desire to take care of themselves at home, the needs of these patients should be identified and understood more deeply, which was one of the reasons for conducting this study. Nurses can estimate the needs of these patients and identify the factors that affect them with the least cost and time. Determining cancer patients’ needs and problems can provide insights into increasing nurses’ knowledge and experience. It can also improve psychological advice and counseling support provided to patients and can help these patients to engage in self-care actions. The results of the present also showed that although the patients were suffering from different types of cancer, they generally expressed similar care needs. Therefore, if the health care team, especially nurses, can accurately identify cancer patients’ needs, they can provide higher quality care. Furthermore, the training given to patients has a great impact on increasing the quality of self-care in patients.

## Data Availability

Not applicable.

## Abbreviations

GLOBOCAN: Global Cancer Observatory.
INPCR: Iranian National Population-based Cancer Registry.
